# Adnexal masses or perineural (tarlov) cysts? Differentiation by imaging techniques: A case report

**Published:** 2017-09

**Authors:** Firoozeh Ahmadi, Farnaz Akhbari

**Affiliations:** *Department of Reproductive Imaging, Reproductive Biomedicine Research Center, Royan Institute for Reproductive Biomedicine, ACECR, Tehran, Iran.*

**Keywords:** Magnetic resonance imaging, Tarlov cysts, Ultrasonography

## Abstract

**Background:**

Perineural cysts formed within the nerve-root sheath at the dorsal root ganglion. They are most commonly located in the arachnoid covering the junction of the dorsal ganglion and nerve root. They are usually asymptomatic, incidental findings, usually located in the lumbar and sacral region of the spinal canal. It is important to consolidate the imaging findings of this rare disease so clinicians can become more clinically relevant in the evaluation of these cysts.

**Case:**

Herein we report a case of perineural cyst misdiagnosed with hydrosalpinx by pelvic ultrasonography and finally diagnosed with magnetic resonance image.

**Conclusion:**

Perineural cyst should be considered, in the presence of bilateral adnexal masses separated from the ovaries in pelvic sonography.

## Introduction

Perineural cysts, which are also known as Tarlov cysts, are fluid-filled sacs on nerves at the spine (1). They are often asymptomatic, but sometimes they have symptoms based on the locations of the cysts along the spine which include: pain, paresthesia, spasticity, hypertonia, muscular dysfunction or weakness, and radiculopathy leading to unnecessary surgery (2, 3).

Perineural cysts are rarely presented as adnexal cystic masses on sonography (4). They were demonstrated as well-defined, smoothly rounded or lobular and avascular cyst, and located in front of the lumbosacral spine. In pelvic sonography, when the cysts are separate from the ovary and immobile on respiration, suspicious to perineuaral cyst arises (5). Ultrasonography, computed tomography (CT) and magnetic resonance imaging (MRI) are useful methods in the diagnosis of Tarlov cyst, but MRI is better advisable for differential diagnosis between gynecological masses and perineural cysts. On MRI, the cyst is usually of cerebrospinal fluid attenuation or signal intensity but may be slightly heterogeneous owing to internal proteinaceous debris (5). 

This rare clinical case report of Tarlov cyst is described here which mimics adnexal masses in order to provide further familiarity of radiologist with differential diagnosis criteria.

## Case report

A 45-year-old virgin female presented to Royan Institute in January 2016 for pelvic sonography. Her chief complaint was a chronic pelvic pain. She had a regular period.

**Figure 1:**
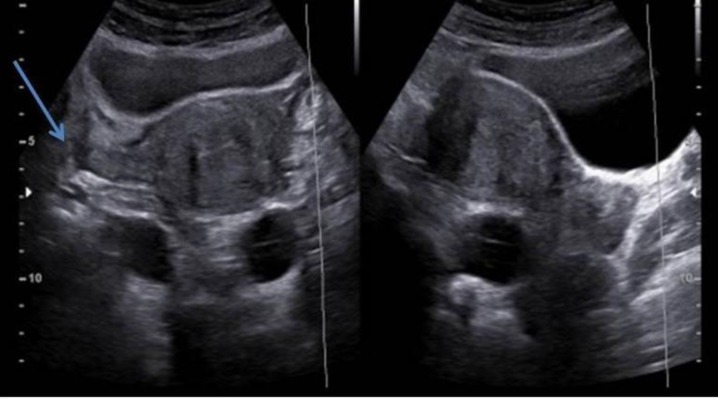
Axial and sagittal ultrasound plane shows normal uterus. Bilateral cystic structure posterior to the uterus is seen and ovaries are demonstrated separately. (Right ovary is detected in the axial image by arrow

Sonography finding showed normal uterus and ovaries. An elongated and irregular bilateral cyst containing free echo fluid at both adnexa, separated from the ovaries was detected (Figure 1). 

**Figure 2:**
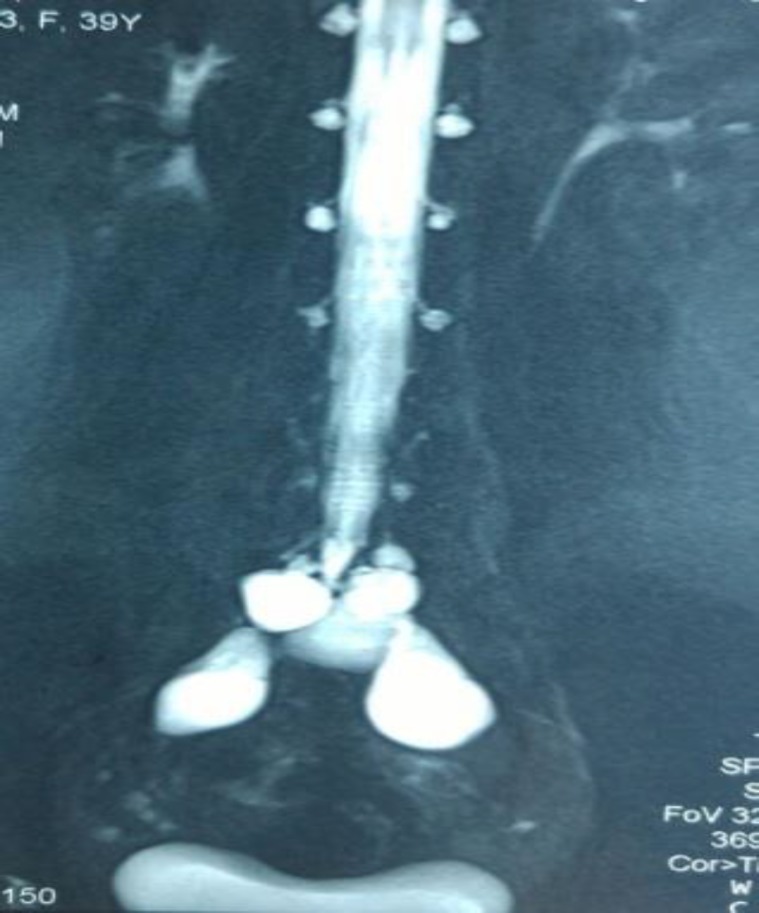
Magnetic resonance myelogram-Axial T1, sagittal T1 and T2 MRI of the lumbosacral region without contrast media. Perineural cyst is present inside the sacral canal at S2- S3 level

The differential diagnostic considerations, based on the sonographic features, included hydrosalpinx or localized fluid due to adhesion, but she had no history of surgery or pelvic inflammatory disease, so these two diagnoses were less considered. Despite antibiotic therapy, the patient suffered pelvic pain and low-back-pain continuously. Follow-up sonography, 3 months later showed persistence structure of cysts. Pelvic MRI was performed in sagittaloblique and axial-oblique planes. The perineural cyst was present inside the sacral canal at the S2-S3 level which has extended to the anterior perisacral space (Figure 2).

The signal characteristics of their contents were the same as those of cerebrospinal fluid. Communication with the thecal sac was well demonstrated and the diagnosis of Tarlov cyst was confirmed. Written consent was taken from the patient for this presentation. Mostly Tarlov cysts do not require any treatment as they are usually asymptomatic in this case patient underwent follow-up sonography.

## Discussion

Perineural cysts were introduced by Tarlov in 1938 (6). They are formed by the arachnoid membrane of the nerve root mostly at the lumbosacral level. Although Tarlov cysts are incidental findings in 4.6-9% of magnetic resonance imaging (MRI) scans, 1% of sacral lesions become symptomatic due to local compression, causing local pain, radiculopathy, and incontinence (7-9). Morphology can vary from a simple rounded cyst to a complex loculated cystic mass. 

Tarlov cysts are uncommon when compared to other neurological cysts, so it is important to recognize them because they can be mistaken by adnexal masses and treated by unnecessary surgery, inappropriately (10). Sonographically, cysts have internal echoes (11-13). The cysts appear as slightly elongated or beaded cystic masses posteriorly. This might present some confusion with a hydrosalpinx, but Tarlov cysts appear less elongated and tubular than a hydrosalpinx. Incomplete septation or “waist sign” which are observed in hydrosalpinx, may not be detected in a Tarlov cyst (14).

Differential diagnosis of ovarian versus extra-ovarian cyst during the sonography is important. With extra-ovarian lesions, the ovaries should be identified as separate structures. Seeing ovarian tissue with follicles around the mass will help confirm an ovarian origin in the patient. Tarlov cysts are fixed and do not move with respiration. The most problematic cases occur when no ovarian tissue is seen around the mass or separate from the mass. It may need to consider both ovarian and extra-ovarian causes such as hematoma, tubo-ovarian abscess, endometrioma, and hydrosalpinx (15).

The most commonly used and effective examination method for Tarlov cysts is MRI (16). Perineural cysts were of high signal intensity on T2-weighted sequences and of low signal intensity on T1-weighted sequences. This case study consolidates key findings, so Ultrasonography could become more clinically relevant in the evaluation of these masses.

## Conclusion

In the pelvic sonography, Tarlov cyst is rare but it should be kept in mind by a radiologist in the differential diagnosis of adnexal masses. When bilateral adnexal masses are detected, searching for separation of ovaries is more important rather than concluded an immediate diagnosis of masses with ovarian origin. 
